# Successive Intramuscular Boosting with IFN-Alpha Protects *Mycobacterium bovis* BCG-Vaccinated Mice against *M. lepraemurium* Infection

**DOI:** 10.1155/2015/414027

**Published:** 2015-09-21

**Authors:** G. G. Guerrero, J. Rangel-Moreno, S. Islas-Trujillo, Ó. Rojas-Espinosa

**Affiliations:** ^1^Unidad Académica de Ciencias Biológicas, Universidad Autónoma de Zacatecas, Avenida Preparatoria, S/N, Colonia Agronomicas, 98066 Zacatecas, ZAC, Mexico; ^2^Division of Allergy, Immunology and Rheumatology, Department of Medicine, University of Rochester Medical Center, 601 Elmwood Avenue, Rochester, NY 14642, USA; ^3^Departamento de Inmunología, Escuela Nacional de Ciencias Biológicas, Instituto Politécnico Nacional, Carpio y Plan de Ayala, Colonia Santo Tomas, 11340 Mexico City, DF, Mexico

## Abstract

Leprosy caused by *Mycobacterium leprae* primarily affects the skin and peripheral nerves. As a human infectious disease, it is still a significant health and economic burden on developing countries. Although multidrug therapy is reducing the number of active cases to approximately 0.5 million, the number of cases per year is not declining. Therefore, alternative host-directed strategies should be addressed to improve treatment efficacy and outcome. In this work, using murine leprosy as a model, a very similar granulomatous skin lesion to human leprosy, we have found that successive IFN-alpha boosting protects BCG-vaccinated mice against *M. lepraemurium* infection. No difference in the seric isotype and all IgG subclasses measured, neither in the TH1 nor in the TH2 type cytokine production, was seen. However, an enhanced iNOS/NO production in BCG-vaccinated/i.m. IFN-alpha boosted mice was observed. The data provided in this study suggest a promising use for IFN-alpha boosting as a new prophylactic alternative to be explored in human leprosy by targeting host innate cell response.

## 1. Introduction

Leprosy is a disease of skin and nerves caused by the intracellular pathogen* Mycobacterium leprae* [1]. It is an ancient disease, which apparently has been eradicated due to the successful multidrug therapy (MDT), reducing the leprosy burden over the years [2, 3]. However, new case detection rates have remained stable over the years at approximately 700,000 new cases per year [3]. In addition, the lack of test to measure asymptomatic* M. leprae* infection in people also hampers more precise assessment of transmission of* M. leprae*. Therefore, present efforts should be addressed not only to the development of improved diagnosis tools [4, 5] but also to immunotherapies. In this sense, a previous work has shown that type I IFNs could be an effective strategy against* M. leprae* infection (Guerrero et al., unpublished results).

The clinical presentation of leprosy has a spectrum that correlates with the type of immune response induced. In the self-healing tuberculoid (T-lep) form, the host immune response is able to effectively combat the pathogen by induction of TH1 type cytokines (bacteria are rare and there are few skin lesions) [6]. In the disseminated lepromatous (L-lep) form, the host immune response fails, resulting in numerous skin lesions characterized by abundant intracellular bacilli. T-lep lesions expressed TH1 cytokines including IFN-*γ*, whereas L-lep lesions are characterized by TH2 cytokines as well as interleukin-10 (IL-10) [6]. Reversal reactions (RRs) represent a shift from the L-lep toward the T-lep form, accompanied by a reduction of bacilli in lesions and enhanced TH1 cytokine responses [6, 7].

Murine leprosy (caused by* Mycobacterium lepraemurium*) (MLM) is a very suitable model to study the clinical immunological correlations of human leprosy due to the striking similitude with human leprosy (caused by* M. leprae*) [6, 7]. In human and murine leprosy, there is a specific depression of cell-mediated immunity (CMI) but no depression of humoral immunity [6, 8]. Although both mycobacteria share striking similarity, there are also differences between them. MLM does not show affinity for peripheral nerves like* M. leprae* [9, 10]. Regardless of the recognition of murine leprosy as a disease different from human leprosy in the early 1930s, the need to create a model for human leprosy still persisted [6, 11]. Both types of mycobacteria are essentially non-cultivable, slow growth in the host, with a doubling time of 1–3 weeks. Experimental infection with small doses of bacteria administered subcutaneously would therefore be expected to closely mimic natural infection. Natural infections usually undergo a stage in which the primary defense barriers are broken, and the initial immune reaction occurs at a peripheral site [12–15]. One important family of infection-induced cytokines is the type I interferons (IFN-I) (*α*/*β*). IFN-I promote differentiation/activation of dendritic cells (DCs) in both human and mice and play an important role in long-term survival of CD8+ T cells in response to specific antigens (Ag) [16–18]. Indeed, plasmacytoid dendritic cells (pDCs) secrete IFN-alpha when stimulated to viral products and differentiate into CD11c+ upon exposure to microbial products [19]. The contribution of the subsets of DCs to the clearance of blood and/or intracellular pathogens is still to be defined. However, recent studies have identified a subset of DCs, tumor necrosis factor-alpha (TNF-alpha)/inducible nitric oxide synthase- (iNOs-) producing DCs (TipDCs), which has been found in the course of viral or bacterial infections [20–22]. Cytokine production, production of reactive oxygen, nitrogen intermediates, and bacterial killing are known effector functions of innate cells including DCs, macrophages, and neutrophils [23, 24]. It has been observed that during Listeria infection there is an increased recruitment of TipDCs in spleen of infected mice, and these DCs were the primary source of TNF-alpha and nitric oxide production [20]. Furthermore, DCs are key linkers of innate and adaptive immune responses by influencing different cell populations, like CD8+ killer T cell responses [25–27]. Therefore, subsets of DCs are potential candidates for immunological interventions, specifically TipDCs, which can protect against recurrent infections [28]. On the other hand, most of the studies concerning the adjuvant activity of IFN-I have been directed toward its antitumoral [29, 30] or antiviral properties [31]. However, the role of type I IFNs in the context of bacterial pathogen remains to be clarified. Indeed, it has been shown that a common IFN-*β* inducible gene program correlates with extent of disease in both leprosy and tuberculosis, suggesting that IFN-*β* is a common factor contributing to pathogenesis in the two distinct mycobacterial diseases. Taking in account this wealth of information, in this work we aimed to investigate the possibility that IFN-*α* boosting of BCG-vaccinated mice might protect mice from intradermal* M. lepraemurium* infection. The obtained data suggest that IFN-alpha boosting of BCG-vaccinated mice could be enhancing iNOS/NO production against* M. lepraemurium* infection.

## 2. Materials and Methods

### 2.1. Antibodies

They were as follows: iNOS/NT/Gr-1, iNOS (goat anti-iNOS, clone M-19, Santa Cruz Biotechnology), Alexa Fluor 568, donkey anti-goat (Invitrogen, A11057), NT (rabbit anti-nitrotyrosine, AB 5411, Millipore), FITC-donkey anti-rabbit (Jackson ImmunoResearch Laboratories, 711-096-152), Gr-1 (biotin-rat anti-mouse Ly6C and Ly6G, clone RB6-8C5, BD Pharmingen), and SA-Cy5 (eBioscience, 19-4317-82).

### 2.2. Animals

Specific pathogen-free BALB/c mice (seven to eight weeks old) were bred and housed in the animal facilities of the ENCB IPN, Mexico City, throughout the entire experiment. All animal experiments were performed with the approval of the Institutional Animal Care and Management Committee to ensure appropriate housing, feeding, and humane handling of the animals.

### 2.3. Microorganisms

The BCG Pasteur strain (isolate 1173P2, World Health Organization, Stockholm, Sweden) was grown as dispersed cultures in Sauton medium for 14 days as described by Menozzi et al., 1998 [32]. The vaccine suspensions were then stored at −80°C until use.* Mycobacterium lepraemurium* non-cultivable strain was isolated from the spleen of mice bearing a 4-month infection via a procedure described by Prabhakaran et al., 1976 [33], followed by the Percoll step described by Draper, 1980 [34]. Briefly, 4 g of tissue was suspended in 20 mL of 0.2 M sucrose and ground in a glass Potter-Elvehjem type homogenizer. The resulting suspension was centrifuged for 20 min at 150 ×g to separate cell debris (Sorvall RV5B, rotor HB4) (Sorvall Instruments, Wilmington, Delaware, USA). Then, 9 mL of the isolated supernatant was overloaded onto 12 mL of 0.3 M sucrose and the tubes were centrifuged at 3500 ×g for 10 min at 4–10°C (Sorvall RC5B). The resulting bacilli-rich pellet was resuspended in 20 mL of 0.2 M sucrose and overlaid, in 9 mL aliquots, into 12 mL of 1.5 M KCL. Then the tube was centrifuged at 4°C for 10 min at 3500 ×g. The bacilli were collected, washed 3 times with phosphate-buffered saline (PBS) at pH 7.4 (PBS is 0.01 M Na/K phosphate, 0,15 M NaCl), and resuspended in 40 mL of a solution containing a mixture of Percoll (3 parts) and 0.1% Tween 80 (7 parts). The suspension was centrifuged at 23000 ×g. The bacillary pellet was collected and washed 5 times with PBS pH 7.4 until the Percoll was completely eliminated. The purity of the bacillary preparation was verified by Ziehl-Neelsen staining. The purified bacillary suspension was prepared in synthetic 7H9 Middlebrook broth-OADC medium (DIFCO, Detroit, MI, USA) (7H9-OADC medium) and quantified via a nephelometric reference curve prepared with known quantities of bacteria. The bacillary suspension was aliquoted and frozen at −20°C until it was ready for use.

### 2.4. Immunizations

Groups of seven- to eight-week-old BALB/c mice were immunized subcutaneously (s.c.) with 5 × 10^5^ colony-forming units (CFUs) of BCG in 200 *μ*L sterile phosphate-buffered saline (PBS) or with 200 *μ*L sterile PBS. One month later, during consecutive days (30, 31, 32 days), each group of mice was boosted intramuscularly (i.m.) with 100 *μ*L of PBS alone or with 100, 200 or 300 UI of IFN-alpha.

### 2.5. *M. lepraemurium* Infection

For the infection experiments, mice were inoculated intradermally with 2 × 10^6^ viable CFUs of MLM in 20 *μ*L of PBS. The mice were then housed in standard conditions and supplied with purified waters and Purina rodent chow chips (Cuautitlan Izcalli, Mexico State., MEXICO). The infections were allowed to proceed for 8 weeks, at which time lesions were totally visible in the skin of infected animals. Animals showed variable degrees of disease progression depending on the treatment given (PBS, BCG, or IFN-alpha).

### 2.6. Enzyme-Linked Immunosorbent Assays (ELISA)

Antibodies in the sera of infected MLM mice were measured by indirect enzyme-linked immunosorbent assays (ELISA). Briefly, 96-well plates (Nunc, NY) were coated overnight at 4°C, with 1 *μ*g of peptidoglycolipids [35] (after evaporation of ethanol in room temperature) [36]. Nonspecific binding was blocked with 3% nonfat milk in PBS for 2 to 3 h. SN was discarded and washed with PBS three times. Addition of 0.1 ml of sera (1 : 1000, 1% non-fat milk) was added to the plate and incubated overnight at 4°C. After incubating samples overnight at 4°C, plates were extensively washed with PBS, and bound antibodies were detected using anti-mouse IgG-HRP (1 : 4000), IgG1-HRP, IgG2a-HRP, IgG2b, or IgG3-HRP, or biotin-conjugated rat anti-mouse IgA monoclonal antibody, followed by streptavidin-horseradish peroxidase conjugate (BD Pharmingen TM). Color reactions were developed by addition of chromogenic substrate, tetramethylbenzidine, in 0.05 M citrate-phosphate buffer (pH 5.2), supplemented with 0.01% H_2_O_2_. The reaction was stopped with 1 M H_3_PO_4_. Optical densities were measured at 450 nm in a microplate ELISA lector (LabSystems Multiskan Plus).

For cytokine measurements in serum, amounts of IFN-*γ*, IL-4, IL-17, IL-6, TNF-alpha, or IL-10 were determined using specific sandwich ELISA kits (PeproTech, Inc), according to the manufacturer's instructions. Data are expressed as the mean ± SEM for each mouse group.

### 2.7. Immunofluorescence

Slides were incubated with goat anti-iNOS, rabbit anti-nitrotyrosine, and biotinylated Gr-1, followed by addition of Alexa Fluor 568, donkey anti-goat, streptavidin Alexa Fluor 488, and Alexa Fluor 488 donkey anti-rat to detect iNOS^+^ cells, NT+ cells (produced by peroxynitrite attack to tyrosine residues), and location of Gr-1^+^ cells. Briefly, 5 *μ*m thick formalin fixed, paraffin sections were hydrated by melting paraffin in a 60°C oven, followed by transfer to xylenes, alcohol, 95% alcohol, and water. After tissue hydration, antigens were unmasked by boiling slides in DAKOCYTOMATION antigen retrieval solution for 30 minutes. After cooling slides down for 10 minutes, they were rinsed with deionized water and transferred to PBS. Nonspecific binding was blocked in 5% normal donkey serum (Jackson ImmunoResearch Laboratories) and 1 *μ*g/mL Fc block (rat anti-mouse CD16/CD32, 2.4G2, BioXcell) in PBS, for 30 minutes at room temperature. After blocking, slides were incubated with primary antibodies in PBS, overnight, at room temperature. Fluorescently labeled, secondary antibodies were added to the slides and incubated for 3 hours at room temperature. Slides were mounted with ProLong Gold antifade with DAPI to visualize nuclei and prevent bleaching. Individual pictures were both taken at 200x magnification with a Zeiss Axioplan Microscope. Inflammatory cell infiltrates, inside the leprosy skin lesion, were outlined with an automated tool of the Zeiss Axiovision software and the average area occupied by inflammatory cells was calculated (*n* = 5 mice per group).

### 2.8. Measurements of NO in the Sera of Mice

In sera from control (healthy mice) and infected mice, NO metabolites (nitrite) were determined using the Griess reaction as described in Miranda et al., 2001. Briefly, sera were diluted 1 : 7 in ethanol to eliminate proteins and/or compound that could interfere with Griess reaction (like S-nitrosothiols or L-arginine derivatives). Precipitated proteins were separated by centrifugation to 3000 rpm/20 min. After, Griess reactive is prepared by mixing Sulfanilamide, 2% p/v in 5% HCl (solution A) with N-1-(naftil) etilendiamine dihidroclorure, 0.1% p/v in 5% HCL (Solution B). The final reaction volume of 200 *μ*L contains 100 *μ*L of mixed sol A+ sol B, 80 *μ*L distilled water, and 20 *μ*L of mouse serum. The samples were analyzed on an ELISA reader at 595 nm. The values were transposed to a standard curve (NaNO_2_ 0.1 mM in a range of 0.9 to 10 *μ*M).

### 2.9. Statistical Analyses

Statistically significant differences among groups were determined by one-way ANOVA. Differences between the means were calculated by Tukey's test. Differences at *P* values < 0.05 were considered statistically significant.

## 3. Results

### 3.1. BCG-Primed Adults Mice Boosted Successively with IFN-Alpha Show an Enhanced Protection against Intradermal* M. lepraemurium* Infection

**Figure 1:**
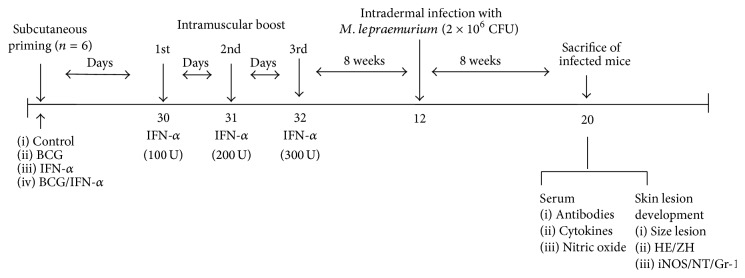
Schematic representation of the prime-boost protocol designed for the study. Adult BALB/c mice were s.c. primed with PBS or 5 × 10^5^ CFUs BCG. Four weeks later, in consecutive days (30, 31, and 32 days), the mice of each group received PBS or 100 UI IFN-*α* i.m. Mice were rested for 10 weeks and then challenged by intradermal route with 2 × 10^6^ *M. lepraemurium*. Eight weeks after challenge, mice were sacrificed and skin lesion development was measured.

**Figure 2:**
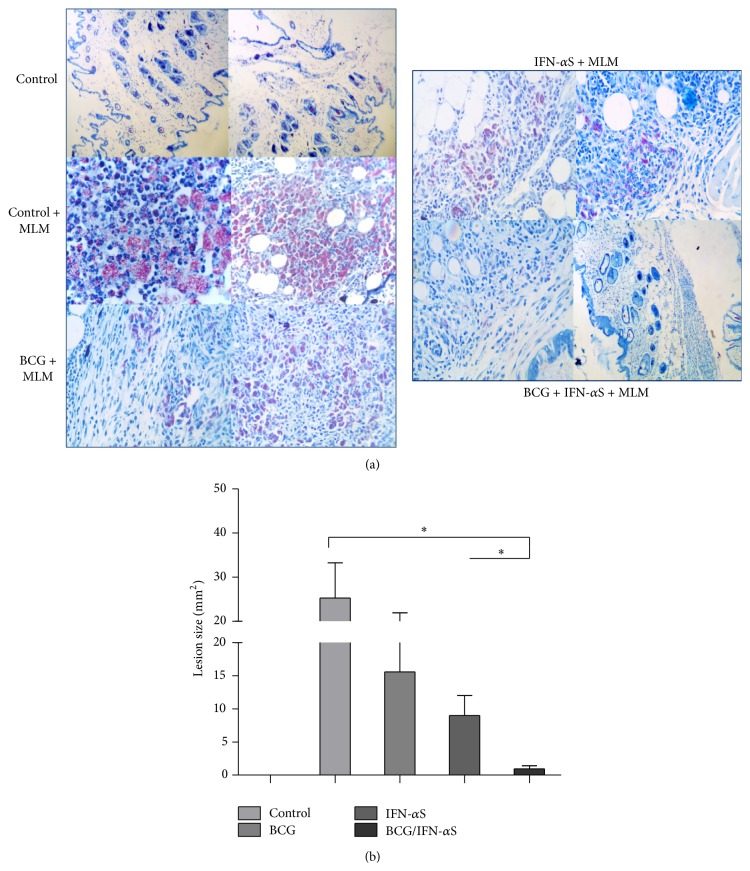
Histopathological appearance of the lesions (representative results) in the skin of BCG-primed/IFN-alpha boosted and infected mice with* M. lepraemurium* ((a) right panel), or BCG-vaccinated/PBS-boosted and infected mice with* M. lepraemurium* ((a) left panel). Sections were stained with Ziehl-Neelsen and Harris'-Hematoxylin (blue) (×40) (a). Graphics of the skin lesion size in MLML infected mice (b). A difference of ^*∗*^ *P* < 0.05 was considered statistically significant.

It has been reported that type I IFNs are potential adjuvants against intracellular pathogens [27, 28, 31]. In this work, IFN-alpha's ability to boost protection in BCG-vaccinated mice against* M. lepraemurium*, using a prime-boost protocol adapted from Guerrero et al., 2015 (unpublished results) (Figure 1), was investigated. Eight weeks after challenge with* M. lepraemurium*, reduction in skin lesion development of infected mice was assessed. Interestingly, BCG-primed/IFN-alpha boosted mice showed a significant decrease in the skin lesion development (2.2 log reduction, 0.97 mm^2^), compared to BCG-primed mice, boosted with PBS (15.6 mm^2^) (Figure 1(b)) (*P* < 0.05) and to control-PBS boosted mice (25 mm^2^). Mice immunized with IFN-alpha alone develop also a similar skin lesion size. These results indicate that successive IFN-alpha boosting of BCG-primed mice enhanced protection against* MLM* infection (Figure 2(a)).

### 3.2. Systemic Antibodies and Cytokine Profile of BCG-Primed/IFN-Alpha Boosted Mice after MLM Challenge

It has been reported that in human and murine leprosy, there is a specific depression of cell-mediated immunity (CMI) but no depression of humoral immunity [6, 7]. Eight weeks after MLM infection, mice were sacrificed and antibody and cytokines were measured in the serum. BCG-vaccinated adult mice, boosted with IFN-alpha by the i.m. route elicited a similar isotype [IgG, 1.34 ± 0.69; IgM, 1.20 ± 0.55; IgA, 1.20 ± 0.41] and all IgG subclasses [IgG1, 1.95 ± 0.68; IgG2a, 1.9 ± 0.77; IgG2b, 1.8 ± 0.56; IgG3, 1.30 ± 0.55], compared to BCG-primed mice boosted with PBS [IgG, 1.1 ± 0.18; IgM, 0.9 ± 0.13; IgA, 0.94 ± 0.19], [IgG1, 1.8 ± 0.07; IgG2a; 1.8 ± 0.20; IgG2b, 1.6 ± 0.31; IgG3, 0.90 ± 0.13] (Table 1). However, IFN-alpha primed mice and i.m. boosted with IFN-alpha induced a higher amount of IgG (1.5 ± 0.21) and IgG1 (2.2 ± 0.22) and a very similar amount of IgM (0.98 ± 0.17), IgA (1.1 ± 0.23), and IgG2a (1.7 ± 0.17), compared to BCG-vaccinated/i.m. IFN-alpha boosted mice (Table 1).

In the other hand, cytokine production was very similar among the different groups of infected mice. Thus, BCG-primed/i.m. IFN-alpha boosted mice and MLM infected mice produced a similar magnitude of Th1 type cytokines, like IFN-*γ* (1460 ± 187 pg/mL) and TH2 type cytokines, namely, IL-4 (1870 ± 451 og/mL) (Table 2) or IL-17 (1128 ± 260 pg/mL), compared to BCG-primed and PBS-boosted mice [IFN-g, 1274 ± 283 pg/mL; IL-4, 1805 ± 149 pg/mL; IL-17, 1062 ± 222 pg/mL]. A similar trend was found for TNF-alpha (1815 ± 467 pg/mL versus 1675 ± 670 pg/mL) and IL-10 (2477 ± 169 pg/mL versus 2060 ± 140 pg/mL). Worthy of notice is the higher amount of IL-4, IL-10, or TNF-alpha compared to the IFN-*γ* or IL-17 quantity (Table 2).

### 3.3. Differential Inducible Nitric Oxide Synthase (iNOS) and Nitrotyrosine (NT) Expression in the Skin Lesion of BCG-Vaccinated/i.m. IFN-*α* Boosted Mice after MLM Challenge

**Figure 3:**
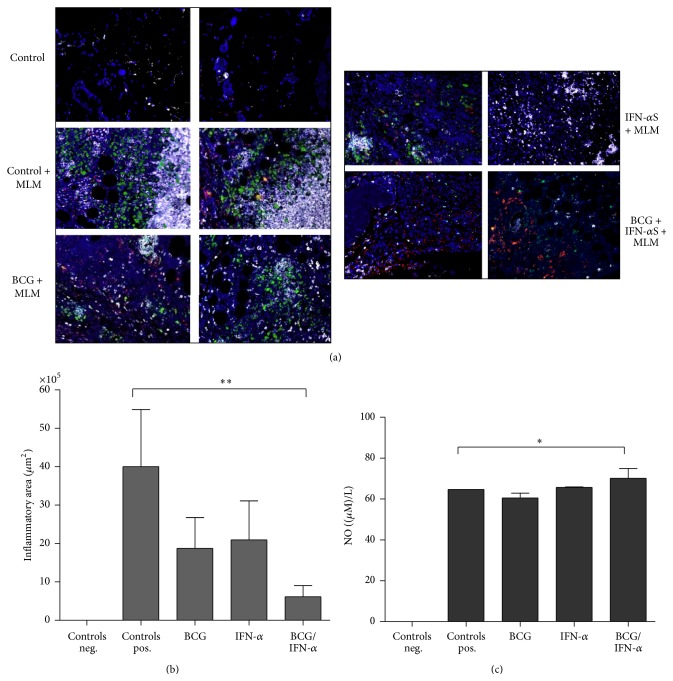
Representative results of the inflammatory area in the granuloma and oxide nitric production in serum of the infected mice with* M. lepraemurium*. Eight weeks after intradermal MLM infection, mice were sacrificed and skin lesions were obtained. Inducible Nitric Oxide Synthase (iNOS, red), NT (green), Gr-1 (White) expression were measured by immunofluorescence (a). Graphics of the inflammatory area (a) and nitric oxide concentration in serum of uninfected, MLM-infected, BCG-vaccinate, IFM-alpha-boosted and BCG-vaccinated/IFN-alpha boosted mice (b) are shown. A difference of ^*∗*, *∗∗*^ *P* < 0.05 was considered significant.

Since a protective IFN-alpha boosting effect was observed in BCG-primed adult mice against challenge with* M. lepraemurium*, we decide to investigate under the settings established for this study the production of antimycobacterial factors (like inducible nitric oxide synthase, iNOS, a proinflammatory protein) associated with protective immunity development [35, 37–41]. Inflammatory area represented by iNOS, nitrotyrosine (NT), and infiltration of granulocytes was measured in the skin lesion of infected mice by immunostaining with fluorescent antibodies iNOS/NT/Gr-1+. A differential ratio of iNOS/NT/Gr-1+ expression was found among BCG-primed/i.m. IFN-alpha boosted mice (iNOS, red; NT, green; and granulocytes, white) and BCG-primed/i.m. PBS-boosted mice (Figures 3(a) and 3(b)) and/or to IFN-alpha administered alone (Figures 3(a) and 3(b)). An small increase in NO production in infected mice was primed with BCG and IFN-alpha, versus control, and/or BCG-vaccinated mice (Figure 3(c)), suggesting a potential effect of the IFN-alpha boosting on the observed iNOS/NT/Gr-1+-ratio and, for instance, in enhancing antimicrobial activities of granulocytes.

## 4. Discussion

In this work, we are showing that IFN-alpha boosting of BCG-primed mice protects against* M. lepraemurium* infection. The observed protection correlated mostly with enhanced iNOs and NO production.

It has been shown that nitric oxide (NO) is a relevant antimycobacterial factor in mouse macrophages and is one of the most efficient bactericidal pathways [35, 37–41]. NO is a product of inducible nitric oxide synthase (iNOS). NO toxicity is greatly enhanced by reacting with superoxide to form peroxynitrite that reacts with many biological molecules [38]. Tyrosine from the proteins is one of the molecules with which NO reacts and the product is nitrotyrosine (NT) [39]. The production of the peroxynitrite and the nitrosylation of proteins might play a role in bacterial killing and also in mediating host injury [38, 39]. iNOS expression has been detected in lesion of murine leprosy at 21 weeks after infection [41] and during the evolution of experimental pulmonary tuberculosis [42]; particularly at early stages of infection, there is also an increased and rapid expression of iNOS by activated macrophages. During this time a high NT production also occurs, with the tubercle bacilli being the most important target of this toxic product. At the late phase of infection (2 and 4 months), iNOS expression declines but NT immunostained cells increase, correlating with an increase in the bacterial burden as well as in tissues damage [42]. It seems that during the early phase of the infection NO and NT participate in the control of the infection, but their high and constant production contribute to host cellular damage during the late phase of infection. Indeed, in our settings of vaccination, BCG-primed/i.m. IFN-alpha boosted mice, at eight weeks after infection, there is an increase of iNOS but a decrease of NT, suggesting a more protective role of iNOS in the healing of mice (mice that develop a very small skin lesion, 0.97 mm^2^ of diameter), comparable to BCG-vaccinated mice/PBS-boosted mice (23 mm^2^). Our data reinforce the observation that iNOS in the murine model of leprosy as well as in pulmonary tuberculosis play an important protective role [37, 42].

On the other hand, several studies had shown that early granulomas in human and mouse leprosy had a proinflammatory environment. In contrast, late granulomas are enriched in anti-inflammatory cytokines [3]. In addition, nitrosylation products and cell alterations were observed in granulomas in the advanced stages of the disease, strengthening observations that NT steadily increased production during the early to late stage of infection can contribute to an anti-inflammatory environment, driven precisely by the bacilli itself, for both the bacillus replication and the diseases progression. Thus, it seems that undervaccination settings used in this study (Figure 1) promoted an environment that did not allow the establishment of the bacilli and skin lesion development [39]. Moreover, it has been reported that after 21 week after infection, macrophage activation is no longer observed and anergy to MLM prevails until 20–24 weeks. In this study, eight weeks after infection, BCG-primed/i.m. IFN-alpha boosted mice showed a very similar antipeptide glycolipid antibody levels of IgG, IgM, and IgA as well as all IgG subclasses analyzed (Table 1), and/or cytokine production (Table 2) compared to BCG-vaccinated/PBS-boosted mice, or control mice (Tables 1 and 2). Despite of this, it is highly possible that humoral response can be directed to other mycobacterial antigens, since it has been shown that humoral and cellular immune responses (Th1 or Th2) could be also important immunological parameters in leprosy [6]. Furthermore, the consequences of type I IFNs signalization on host outcome can be either protective or damaging. Type I IFNs induction could affect bacterial clearance processes including cell migration, apoptosis, inducible nitric oxide synthase expression, and secretion of chemokines and cytokines [43–45]. From the literature it is well accepted that protective immunity against many intracellular bacteria (like* M. leprae*) depends on type I helper (TH1) T cell responses [43] in particular the production of the type II interferon (IFNs), like IFN*γ*, which can activate antimicrobial mechanism responses [43–45]. On the other hand, TipDCs (TNF-alpha/iNOS/NO producers) could be a potential immunotherapeutic target since it has been described that partial blocking of these cells protects against bacterial infections by priming CD8+ killer T cells [20–22].

Collectively, the data of this study show under a protocol of IFN-alpha boosting and BCG-vaccination of adult mice that it might be possible to target innate immunity (macrophages/neutrophils or TipDCs, iNOS/NO producers) (Figures 3(a)–3(c)), which can contribute to limit bacterial growth and therefore skin lesion development (Figure 2(a)). Altogether, our study provides a new prophylactic prime-boost protocol based on successive IFN-alpha boosting of BCG-priming mice that could be explored in human leprosy.

## Figures and Tables

**Table 1:** Antibody response in BALB/c mice with the BCG-priming/IFN-alpha boost after *M. lepraemurium *challenge.

Vaccination group	IgM	IgG	IgG1	IgG2a	IgG2b	IgG3
Control	0.87 ± 0.09	1.10 ± 0.12	1.70 ± 0.34	1.10 ± 0.22	1.30 ± 0.18	0.87 ± 0.15
BCG	0.90 ± 0.13	1.10 ± 0.18	1.80 ± 0.07	1.80 ± 0.20	1.60 ± 0.31	0.90 ± 0.13
IFN-*α*	0.98 ± 0.17	1.50 ± 0.21	2.20 ± 0.22	1.70 ± 0.17	1.50 ± 0.16	1.14 ± 0.05
BCG-IFN-*α*	1.20 ± 0.55	1.34 ± 0.69	1.95 ± 0.68	1.90 ± 0.77	1.82 ± 0.56	1.30 ± 0.55

Eight weeks after infection with MLM, BCG-primed/i.m. IFN-alpha boosted mice were sacrificed. Levels of antiglycolipid-1 isotype and all IgG subclasses Abs were measured in serum and determined by immunoenzymatic assay ELISA. Values are expressed as OD 450 nm and represent median ± SEM of duplicates.

**Table 2:** Cytokine profile in BALB/c mice with BCG-priming/IFN-alpha boost after *M. lepraemurium *challenge.

Vaccination group	IFN-*γ*	IL-4	IL-17	TNF-*α*	IL-10
SERUM					
Control	912 ± 102	1360 ± 406	762 ± 58	511 ± 147	1826 ± 318
BCG	1274 ± 283	1805 ± 149	1062 ± 222	1675 ± 670	2060 ± 140
IFN-*α*	1076 ± 233	1683 ± 211	999 ± 177	1485 ± 560	2397 ± 514
BCG-IFN-*α*	1460 ± 187	1870 ± 451	1128 ± 260	1815 ± 467	2477 ± 169

Eight weeks after infection with MLM, BCG-primed/IFN-alpha boosted mice were sacrificed. Levels of cytokines were measured in serum and determined by immunoenzymatic assay ELISA (PeproTech, Inc). Values are expressed to pg/mL and represent median ± SEM of duplicates.
